# Susceptibility of Pigs and Chickens to SARS Coronavirus

**DOI:** 10.3201/eid1002.030677

**Published:** 2004-02

**Authors:** Hana M. Weingartl, John Copps, Michael A. Drebot, Peter Marszal, Greg Smith, Jason Gren, Maya Andonova, John Pasick, Paul Kitching, Markus Czub

**Affiliations:** * Canadian Science Centre for Human and Animal Health, Winnipeg, Manitoba, Canada

**Keywords:** severe acute respiratory syndrome, coronavirus, experimental infection, swine, chicken

## Abstract

An outbreak of severe acute respiratory syndrome (SARS) in humans, associated with a new coronavirus, was reported in Southeast Asia, Europe, and North America in early 2003. To address speculations that the virus originated in domesticated animals, or that domestic species were susceptible to the virus, we inoculated 6-week-old pigs and chickens intravenously, intranasally, ocularly, and orally with 10^6^ PFU of SARS-associated coronavirus (SARS-CoV). Clinical signs did not develop in any animal, nor were gross pathologic changes evident on postmortem examinations. Attempts at virus isolation were unsuccessful; however, viral RNA was detected by reverse transcriptase-polymerase chain reaction in blood of both species during the first week after inoculation, and in chicken organs at 2 weeks after inoculation. Virus-neutralizing antibodies developed in the pigs. Our results indicate that these animals do not play a role as amplifying hosts for SARS-CoV.

An outbreak of severe acute respiratory syndrome (SARS) in humans, associated with a new coronavirus (SARS-CoV), has been reported in Southeast Asia, Europe, and North America (*1*–*3*). According to the World Health Organization, SARS affected more than 8,200 people worldwide and killed more than 700. The sequence analysis of SARS-CoV suggests that it is substantially distinct from all other known coronaviruses (*1*,*2*). Based on the nucleotide sequence, the virus is speculated to have evolved and been maintained in an animal host. However, no conclusive data have been presented to date on a possible reservoir for this virus. Our study aimed to address the role of domestic animals in the outbreak, both from the public health perspective (as a potential source of virus for human infections) and the animal health perspective. A potential susceptibility of domestic species to SARS-CoV would have major implications on the management of livestock operations worldwide.

We have experimentally inoculated chickens and swine. Both species are natural hosts for a number of viruses from the same family as SARS-CoV (*Coronaviridae*). The infectious bronchitis virus of chickens, although distinct, groups genetically most closely with SARS-CoV (*1*,*2*). Swine can host several coronaviruses (hemagglutinating encephalomyelitis virus, transmissible gastroenteritis virus [TGEV], and porcine respiratory coronavirus [PRCV]). In addition, continuous cultures of porcine turbinate cells (PT-K75) and primary chicken embryo epithelial kidney cells supported SARS-CoV replication.

## Material and Methods

### Animals

Six 4-week-old crossbred pigs were kept for 2 weeks to acclimatize before being inoculated. The pigs, obtained from a high health status herd (Sunnyside Colony LTD, Sunnyside, Manitoba), had preexisting antibodies against PRCV, likely of maternal origin (*4*), which decreased during the experiment, as determined by competitive enzyme-linked immunosorbent assay performed by the Veterinary Services Branch of Manitoba Agriculture and Food.

Six-week-old, nonvaccinated, specific-pathogen-free chickens (White Leghorn), obtained from ADRI Nepean (Nepean, Ontario) were kept for 3 days to acclimatize before inoculation. They were housed in chicken isolators inside a biosafety level 4 (BSL4) animal cubicle. Animal housing and all animal manipulations were approved by the Animal Care Committee of the Canadian Science Centre for Human and Animal Health and met the Canadian Council on Animal Care guidelines.

### Virus

SARS-CoV was plaque purified from a human isolate (Tor 3) on Vero E6 cells by using the plaquing method we describe in the SARS-CoV antibody detection section. Virus stock for animal inoculation was prepared and titrated on Vero V76 cells. Virus replication in Vero E6, Vero V76, and PT-K75 cells was compared by employing the following plaque assay: an aliquot of each virus dilution was added in duplicate onto cell monolayers in 12-well plates (Costar, Corning, NY). Virus inoculum was then incubated on cells for 1 h at 37°C, 5% CO_2_, and removed. The cells were overlayed with 2% carboxymethyl-cellulose (Sigma, St. Louis, MO)/ Dulbecco modified Eagle medium (DMEM) (Wisent, St. Bruno, Quebec), and incubated at 37°C, 5% CO_2,_ for 4 days. At the end of the incubation, the cells were fixed with 4% formaldehyde and stained with crystal violet. The experiment was repeated twice.

### Cells

Vero E6 and Vero V76 (ATCC) cells were maintained in 10% fetal bovine serum (FBS)/DMEM medium (Sigma). Porcine turbinate PT-K75 (ATCC) cells were maintained in 10% FBS/ DMEM (Wisent). Quail QT-35 (ATCC) cells were maintained in 10% FBS/MEM-alpha medium (Mediatech Cellgro, Herndon, VA).

For the preparation of primary chicken embryo kidney epithelial cells (CEKEC), kidneys were harvested from 18-day-old chicken embryos and digested with 3 U of pronase (Sigma)/mL in citrate buffer (1.5 mM KCl, 27 mM sodium citrate, 8 mM KH_2_PO_4_, 5.6 mM Na_2_HPO_4_, pH 7.3) by repeated incubation for 2 min at 37°C with stirring. Cells were collected into fetal bovine serum and washed extensively with phosphate-buffered saline before being seeded into 24-well plates (Costar). Cells were seeded at a density of 10^6^cells/cm^2^ in 1% FBS/Williams medium (Sigma) for the virus isolation. The cell suspension contained about 95% epithelial cells and 5% fibroblasts after 24 h of incubation, as previously determined by immunofluorescent assay; markers for epithelial cells (cytokeratin) and fibroblasts (vimentin) were detected.

### Experimental Infection

The preimmune serum from chickens and pigs was collected 2 days before inoculation. Six-week-old pigs were inoculated simultaneously by four routes, intravenously, intranasally, ocularly, and orally, with 2 x 10^6^ PFU of SARS-CoV per pig. Six-week-old chickens were inoculated by the same routes with 10^6^ PFU of SARS-CoV per chicken. Three pigs and three chickens were mock inoculated and served as negative controls. Both species were divided into two groups, and blood, nasal (nares), throat, and rectal (cloacal) swabs were collected on alternate days, starting at 2 days after inoculation (dpi) and ending at 7 dpi. On days 6, 7, 13, 14, 15, and 16 after inoculation, one pig and one chicken per day were euthanized. In addition to swabs and blood, samples from lung, trachea, liver, heart, spleen, kidney, tonsil (pig), and jejunum were collected at postmortem examination. All experimental work was carried out in BSL4 containment.

### Virus Isolation

Virus isolation from porcine samples was attempted on Vero V76 and porcine turbinate cells PT-K75, seeded at a density of 2 x 10^5^ cells/cm^2^ in 12-well plates (Costar) 24 h before inoculation. Samples were tested in duplicate twice, by plaque assay (described in Virus section) and monitoring cytopathic effect (CPE), followed by reverse transcriptase–polymerase chain reaction (RT-PCR) to detect virus replication. In addition, virus isolation from chicken samples was attempted on chicken embryo epithelial kidney cells, seeded at a density of 10^6^ cells/cm^2^ in 24-well plates (Costar), using CPE format followed by RT-PCR.

The tissues were ground in a MiniMix blender (Topac, Hingham, MA) to prepare a 10% w/v suspension in Dulbecco’s PBS (Sigma) supplemented with antimicrobial drugs and stood for 1 h in the antimicrobial mix (streptomycin/vancomycin/nystatin/gentamycin). The suspension was centrifuged at 2000 x *g*, 4°C, 20 min. The supernatant was diluted 10-fold in the corresponding media for the individual cell types, and 400 μL (in duplicates) was incubated on cells for 1 h at 37°C, 5% CO_2_. The inoculum was then removed and replaced with the appropriate media, supplemented with 5% FBS (Vero and PT-K75 cells) or 1% FBS (CEEKC). Plates were incubated for 5 days at 37°C, 5% CO_2_. Isolation from blood and swabs was performed as for tissues without the homogenization step. The sensitivity of virus isolation was determined by spiking negative control lung tissues from one chicken and one pig with virus inoculum before homogenization, titrating out the samples on Vero E6 and Vero V76 cells, and comparing the titers to the inoculum titer, using plaque assay described in the virus section.

### RT-PCR

RNA was extracted from blood and tissue samples with the TriPure Extraction kit (Roche Diagnostics, Indianapolis, IN). Three sets of primers were used in a one-step RT-PCR assay employing the Qiagen OneStep RT-PCR kit (Qiagen, Mississauga, ON): 1. NML polymerase primers: forward primer CAG AGC CAT GCC TAA CATG and reverse primer AAT GTT TAC GCA GGT AAG CG were used in the RT-PCR reaction (50°C for 30 min, 95°C for 15 min, followed by 50 cycles of 94°C for 15 s, 50°C for 30 s, 72°C for 30 s with 7-min extension at 72°C). The 389-nt amplicon is located within the RNA-dependent RNA polymerase gene (ORF 1b). 2. Nucleocapsid (N) primers: forward primer ATA ATA CTG CGT CTT GGT TC and reverse primer TGG CAA TGT TGT TCC TTG AG were used under the same reaction conditions as the first set of primers, yielding a 364-base pair (bp) long amplicon. 3. BNI polymerase primers and RT-PCR conditions were developed at the Bernhard-Nocht Institute for Tropical Medicine, Hamburg, Germany, by C. Drosten, and published on their Web site on March 26, 2003: BNI OUT S2: ATG AAT TAC CAA GTC AAT GGT TAC (forward); BNI OUTAS: CAT AAC CAG TCG GTA CAG CTA C (reverse). The RT-PCR conditions were: 30 min at 50°C, 15 min at 95°C, followed by 50 cycles of 95°C for 10 s, 56°C for 10 s, 72°C for 20 s, and completed at 72°C for 7 min, yielding an amplicon of 195 bp.

The Qiagen OneStep RT-PCR kit was also used for the two-step RT-PCR with the following modifications: the template was incubated at 50°C for 30 min only with forward N primer followed by the incubation at 95°C for 15 min to inactivate the reverse transcriptase. Residual single-strand RNA template was removed by digestion at 37°C for 20 min with 2 U of Rnase H (Invitrogen, Burlington, ON). After both forward and reverse N primers and the Platinum Pfx DNA polymerase (Invitrogen) were added, the DNA synthesis was completed by using the above conditions for the N primers in a one-step RT-PCR. Randomly selected amplicons were sequenced with the respective primers to verify the identity of the bands. Sensitivity of the individual primer sets used in the RT-PCR assays was tested by spiking negative control lung tissues from chicken and pig with virus inoculum before homogenization, titrating out the homogenate, and running the RT-PCRs in parallel on the same RNA extracts.

### SARS-CoV Antibody Detection

Porcine serum collected before inoculation and during the final bleed was tested for antibodies against SARS by a standard plaque reduction neutralizing test, as previously described (*5*). Briefly, mixtures of pre-titered (100 PFUs) SARS-CoV and serial twofold dilutions of animal sera were incubated at 37°C for 1 h and added to 6-well plates containing Vero E6 cell monolayers. After a 37°C incubation for 1 h, a nutrient-agar overlay was added, and the plates were placed in a CO_2_ incubator for approximately 3 days. A second overlay, which contained neutral red as a vital stain, was then added. Plates were then checked periodically over the next few days for plaque formation. The highest serum dilution, which produced a plaque reduction of at least 90%, was defined as the titration end point.

### Porcine Serum Cross-Reactivity with TGEV/PRCV

Serum samples collected on the pre-inoculation bleed and terminal bleed were tested for neutralizing antibodies against SARS and TGEV/PRCV by using microtiter CPE blocking assay. Each of the above viruses was diluted to 100 50% tissue culture infective dose (TCID_50_)/well, mixed with doubling serial dilutions of test serum beginning at 1:5 (giving the first serum dilution 1:10), and incubated for 1 h at 37°C. The virus-serum mixtures were then added to 96-well microtiter plates (Costar) containing overnight confluent monolayers of Vero V76 cells or PT-K75 cells, for the SARS or TGEV CPE-blocking assay, respectively. The results were read after 3 days of incubation at 37°C, 5% CO_2_.

## Results

Preliminary tests to establish a sensitive cell system for virus replication were performed before animal inoculation and virus isolation. SARS-CoV replicated in Vero E6, Vero V76, and PT-K75 approximately to the same titer. QT-35 did not replicate SARS-CoV. Although CEKEC did not show any CPE, the virus replicated in those cells up to the approximate titer of 10^6^, based on positive RT-PCR results on lysed cells and cell culture supernatant harvested 54 h after inoculation.

### Animal Inoculation

Neither clinical disease nor gross pathologic changes were observed in chickens or pigs. Repeated attempts to isolate SARS-CoV from swabs, blood, and organs on Vero V76 had negative results. No significant (drop in titer within 1 log) impact of tissue processing on the infectivity of virus during virus isolation was observed by using lung tissues from one control chicken and one pig under control conditions. The tissue was spiked with SARS-CoV before homogenization, and virus recovery was compared to the correspondingly diluted inoculum on Vero E6 and Vero V76 cells (Table 1). Additional attempts at virus isolation were carried out on PT-K75 cells and, with chicken samples, on chicken embryo kidney epithelial cells. The results were again negative, as confirmed by RT-PCR on the inoculated cells.

**Table 1:** Relative sensitivity of virus isolation and RT-PCR in tissue samples spiked with the SARS virus before homogenization^a,b^

Tissue samples	Virus isolation (PFU/100 μL)		RT-PCR (100 μL)		
	Vero E6	Vero V76	NML primers	N primers	BNI primers
Virus-spiked control	‑5.8	‑6	‑8	‑10	‑10
Chicken lung	‑5.6	‑5.75	‑7	‑9	‑9
Pig lung	‑5.5	‑5.7	‑7	‑9	‑9

^a^RT-PCR, reverse transcriptase–polymerase chain reaction; SARS, severe acute respiratory syndrome; N, nucleocapsid. ^b^The highest dilution in which the virus or the RNA were detected is given in log_10_^.^

RT-PCR assays were undertaken by using three sets of primers, one developed at the National Microbiology Laboratory during the investigation of the Toronto outbreak of SARS, which targeted the polymerase gene, a second (BNI) set also within the polymerase gene, and the third set targeting the nucleocapsid gene region. Due to the presence of 3′-coterminal nested mRNAs and genomic RNA (*6*,*7*) during coronavirus replication, nucleocapsid RT-PCR was expected to be more sensitive in samples containing replicating virus. The originally used NML primers were less sensitive than the other two sets of primers (BNI pol and N), and the samples were retested with these two sets of primers. Sensitivity of the RT-PCR employing the individual primer sets is illustrated in Table 1, as determined by using negative control lung tissues spiked with SARS-CoV. RT-PCR with the N and the BNI primers detected viral RNA equivalent to approximately 10^-3/-4^ PFU.

RT-PCR amplicons were detected in blood samples from chickens and pigs at 2 (pig 9, chickens 114 and 115) and 3 (pigs 10, 11, 12, chickens 116, 117, 118) dpi using the NML polymerase primers. Positive results using a two-step RT-PCR assay, aimed at detecting negative strands of viral RNA, indicated that replicating virus was present in the above positive pig and chicken blood samples (Figure). By using N primers and the BNI primers, viral RNA was detected in blood of all inoculated chickens up to 7 dpi and in chicken 113 at 15 dpi (Table 2).

**Figure:**
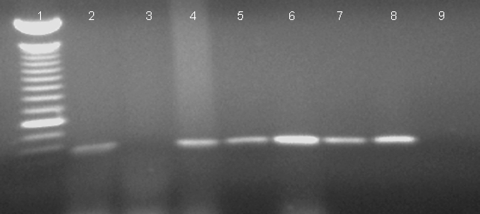
Amplification of severe acute respiratory syndrome–associated coronavirus (SARS-CoV) RNA in chicken blood, using one-step and two-step reverse transcriptase–polymerase chain reaction (RT-PCR) with nucleocapsid primers. Lane 1: 100-bp ladder, the bright band representing 600 bp; Lane 2: chicken 115, 2 days postinocuation (dpi), one-step RT-PCR; lane 3: chicken 115, 2 dpi, two-step RT-PCR (detecting negative-strand RNA); lane 4: chicken 117, 3 dpi, one-step RT-PCR; lane 5: chicken 117, 3 dpi, two-step RT-PCR; lane 6: chicken 115, 4 dpi, one-step RT-PCR; lane 7: chicken 115, 4 dpi, two-step RT-PCR; lane 8: SARS-CoV–infected cells; lane 9: negative control.

**Table 2:** RT-PCR on blood samples from chickens using different primer sets^a^

dpi	Chicken no.	Primers		
		NML pol	N	BNI pol
2	113	-	+	+
	114	+	+	+
	115	+	+	+
3	116	+	+	+
	117	+	+	+
	118	+	+	+
4	113	-	+	+
	114	-	-	+
	115	-	+	+
5	116	-	+	+
	117	-	+	+
	118	-	-	-
6	113	-	+	+
	114	-	+	+
	115	-	+	+
7	116	-	+	+
	117	-	-	-
	118	-	-	-
13	114	-	-	-
14	116	-	-	-
15	113	-	+	+
16	117	-	-	-

^a^RT-PCR, reverse transcriptase–polymerase chain reaction; dpi, days postinfection; pol, polymerase; N, nucleocapsid.

No viral amplicons were generated from any of the harvested organs or swabs when the NML polymerase primers were used; however, the N primers yielded amplicons from spleens of two pigs at days 7 and 13 after inoculation, and in a number of chicken organs. Lung, kidney, and trachea were positive in some birds at 13 to 16 dpi, while liver, spleen, and jejunum samples were all negative. These results were confirmed with BNI polymerase primers (Table 3). Sequence analysis of selected amplicons confirmed the SARS-CoV nucleotide sequence.

**Table 3:** Summary of RT-PCR results on chicken tissues^a^

Chicken no.	dpi	Lung		Trachea		Heart		Liver		Spleen		Kidney		Jejunum	
		N	BNI	N	BNI	N	BNI	N	BNI	N	BNI	N	BNI	N	BNI
115	6	-	-	-	-	-	-	-	-	-	-	-	-	-	-
118	7	-	-	-	-	-	-	-	-	-	-	-	-	-	-
114	13	+	+	+	+	-	-	-	-	-	-	+	-	-	-
116	14	-	-	-	-	-	-	-	-	-	-	-	-	-	-
113	15	-	-	-	-	-	-	-	-	-	-	-	-	-	-
117	16	+	-	+	-	-	-	-	-	-	-	+	+	-	-

^a^RT-PCR, reverse transcriptase–polymerase chain reaction; dpi, days postinfection; N, nucleocapsid.

No SARS-CoV–neutralizing antibodies (90% reduction of virus plaques on Vero E6 cells) were detected in prebleedings from pigs and chickens. The preexisting antibodies against PRCV/TGEV in pigs did not neutralize SARS-CoV and decreased during the experiment. Neutralizing antibody against SARS-CoV developed in pigs, with titers ranging from 1:10 to 1:160 at the time of euthanasia. The SARS antibody titers corresponded for both types of virus neutralization tests (the macrotiter plaque reduction assay and the microtiter CPE blocking assay). Table 4 summarizes the changes in SARS- and TGEV-neutralizing antibodies in pigs during the course of the experiment. No antibodies >1:10 were detected in chicken serum samples on the final bleed.

**Table 4:** Overview of virus neutralization titers for pig preimmune and immune sera against SARS-CoV and TGEV^a^

Pig no.	Pre-inoculation bleed serum antibody titer			Final bleed serum antibody titer		
	VNT TGEV	VNT SARS	PRNT TGEV	VNT TGEV	VNT SARS	PRNT SARS
7	0	0	0	0	20	10
8	20+	0	0	20	320	160
9	10	0	0	0	160	80
10	20	0	0	10	80+	80
11	10	0	0	0	40	40
12	20	0	0	0	80	80

^a^Determined by microtiter virus neutralization test (VNT) and plaque reduction neutralization test (PRNT). SARS-CoV, severe acute respiratory syndrome-coronavirus; TGEV, transmissible gastroenteritis virus.

## Discussion

After the experimental exposure of chickens and pigs to SARS-CoV, we detected coronavirus RNA in blood and several tissues from both species starting at 2 days after inoculation. Clearance of low or nonreplicating intravenous inoculum from blood, including the viral RNA, occurs rapidly in a number of viruses (*8*,*9*). In light of the typical clearance rates and the estimated initial virus load of 5 PFU/100 μL (porcine blood), the detection of RNA, corresponding to a minimum of 10^-3^ PFU/ 100 μL, in blood at 48 h after inoculation is likely not due to a nonreplicating residual virus inoculum. Our data suggest that pigs and chickens of the age used in the experiment were infected with SARS-CoV and, to a very limited degree, supported virus replication. The unsuccessful attempts at virus isolation could be explained by a very low rate of virus replication perhaps combined with loss of infectivity during the sample collection and processing. Although the observed decrease in virus recovery (virus spiked control samples) is not significant, it may have played a role in case of the low virus load. The intravenous route likely does not represent the route of infection in a field situation, and the question of possible natural infection of chickens and pigs with SARS-CoV remains open.

Neutralizing antibodies against SARS-CoV developed in the pigs within 2 weeks of inoculation. These antibodies did not cross-react with TGEV/PRCV in a TGEV neutralization assay (PRCV and TGEV are indistinguishable in the virus neutralization assays) (*10*). Likewise, the preinoculation serum samples with the highest TGEV-neutralizing antibodies did not neutralize SARS-CoV, and the neutralizing antibodies against TGEV decreased as the SARS antibody titers increased. Based on the serum neutralization tests, TGEV/PRCV and SARS-CoV do not appear to be antigenically closely related, an observation supported by the initial genomic analysis (*1*,*2*). The cross-neutralization with TGEV/PRCV was initially a concern after the publication of immunohistochemical assays on SARS-CoV–infected cells (*11*). Since virus-neutralizing antibodies often take approximately 3 weeks to develop in chickens, no conclusions were made with regard to the low or absent antibody titers in their sera at 2 weeks after inoculation. In conclusion, the limited extent of virus replication as indicated by RT-PCR, the failure to isolate the virus, and the lack of virus shedding indicate that neither pigs nor chickens are likely to play a role as an amplifying host.
